# Role of novel renal biomarkers in predicting the prognosis of patients with chronic kidney disease: a prospective cohort study

**DOI:** 10.55730/1300-0144.6179

**Published:** 2026-01-19

**Authors:** Raghavan PADMANABHAN, Kaviya MANOHARAN, Melina SAHAY, Pooja BHUJANGARAO, Luxitaa GOENKA, Solai PRIYA, Kumarasamy SUBRAMANIYAN, Damal Kandadai SRIRAM, Melvin GEORGE

**Affiliations:** 1 Department of Nephrology, Faculty of Medicine and Health Science, SRM Institute of Science and Technology, Kattankulathur, India; 2 Centre for Clinical Pharmacology, Faculty of Medicine and Health Science, SRM Institute of Science and Technology, Kattankulathur, India; 3 Department of Clinical Research, Hindu Mission Hospital, West Tambaram, Tamil Nadu, India; 4 Department of General Medicine, Faculty of Medicine and Health Science, SRM Institute of Science and Technology, Kattankulathur, Tamil Nadu, India; 5 Department of Diabetology and Endocrinology, Hindu Mission Hospital, West Tambaram, Tamil Nadu, India

**Keywords:** Lipocalin-2, receptor for advanced glycation end products, RAGE, tissue inhibitor of metalloproteinase 1, TIMP-1, osteopontin, trefoil factor 3, TFF-3, chronic kidney disease

## Abstract

**Background/aim:**

Several biomarkers have been assessed for the diagnosis of chronic kidney disease (CKD). However, limited data are available regarding their ability to predict mortality in CKD. The aim of this study was to assess the ability of lipocalin-2, receptor for advanced glycation end products (RAGE), tissue inhibitor of metalloproteinase 1 (TIMP-1), osteopontin, and trefoil factor 3 (TFF-3) in predicting the prognosis of patients with CKD.

**Materials and methods:**

We included patients with CKD as defined by an estimated glomerular filtration rate of <60 mL/min/1.73 m^2^ of either sex who were above the age of 18 years. Patients with a history of acute-on-chronic kidney disease were excluded. The novel markers of interest were estimated from patients’ plasma samples using the multiplex enzyme-linked immunosorbent assay. These patients were followed for 1 year to assess mortality.

**Results:**

The median (with interquartile range) plasma concentrations of lipocalin-2, RAGE, TIMP-1, osteopontin, and TFF-3 were 56.9 (44.72–64.18) ng/mL, 7.1 (4.92–9.56) ng/mL, 46.44 (33.52–47.56) ng/mL, 60.2 (98.9–167.61) ng/mL, and 4.87 (8.03–15.65) ng/mL, respectively. A combined receiver operating characteristic curve was plotted to determine the ability of the novel renal biomarkers to predict mortality in patients with CKD. The cutoff values for lipocalin-2 and RAGE for predicting mortality were 62.48 ng/mL with sensitivity and specificity of 86% and 78% (area under the curve: 0.814; p = 0.007) and 8.5 ng/mL with sensitivity and specificity of 71% and 72% (area under the curve: 0.738; p = 0.04), respectively.

**Conclusion:**

Biomarkers including lipocalin-2 and RAGE were assessed in this study and were found to have good predictive value for mortality outcomes in CKD. Future studies should establish the relationship between these novel renal biomarkers and the progression of CKD.

## 1. Introduction

Chronic kidney disease (CKD) is a syndrome defined by structural abnormalities and decreased kidney function with an estimated glomerular filtration rate (eGFR) of <60 mL/min/1.73 m^2^ [1], persistent urinary abnormalities, and impaired excretory renal function due to loss of nephrons [2]. It is a noncommunicable chronic disease with an increasing general population prevalence currently ranging from ~5% to 13% worldwide. The prevalence of CKD was found to be 785 per million in the Indian population [3]. By 2040, CKD is anticipated to rank as the fifth leading cause of years of life lost worldwide [4]. Some of the major causes of mortality in these cases include high blood pressure, diabetes, and glomerulonephritis [5]. The complications of CKD are accelerated by the risk of cardiovascular diseases [6]. Kidney dysfunction is evident with changes in the output and quality of urine, edema, and hypertension, accompanied by elevated levels of serum creatinine. Previous studies have focused on eGFR values based on serum creatinine concentrations and alternative prognostic biomarkers are still needed [7]. Novel biomarkers are currently being studied extensively to support diagnostic and prognostic processes for CKD. Quantifying biomarkers that have relationships with the disease pathway would further support the stratification of patients with CKD.

Tissue inhibitor of metalloproteinase 1 (TIMP-1) has a direct antiapoptotic function and also exerts indirect activity via matrix metalloproteinase (MMP) inhibition pathways [8]. Receptor for advanced glycation end products (RAGE) is a signal transduction receptor that binds advanced glycation end products, certain members of the S100/calgranulin family, high mobility group box 1 (HMGB1), advanced oxidation protein products, and amyloid (β-sheet fibrils) [9]. Lipocalin-2 or neutrophil gelatinase-associated lipocalin (NGAL) is a protein secreted from renal tubular cells as a response to injury in cases of renal disease [10]. Osteopontin, present in the thick ascending limb of the loop of Henle, is overexpressed in cases of proteinuria and in clinical scenarios associated with decreased creatinine clearance [11]. Trefoil factor 3 (TFF-3) belongs to a group of peptides that participate in cancer metastasis pathways and is also found to be elevated in CKD [12]. The value of these biomarkers in predicting the prognosis of patients with CKD has not been studied in depth. Therefore, the present study was planned with the aim of investigating the roles of lipocalin-2, RAGE, TIMP-1, osteopontin, and TFF-3 as potential novel biomarkers for predicting mortality in patients with CKD.

## 2. Materials and methods

This prospective cohort study was carried out after obtaining approval from the relevant hospital ethics committees (registration numbers: 1391/IEC/2018 and HMH/IEC/2019/EA46). Study participants, all of whom provided written informed consent, were screened and recruited from the hospital’s intensive care unit and the ward of the Nephrology Department in line with the inclusion and exclusion criteria. We included patients of either sex who were ≥18 years of age and diagnosed with CKD with an eGFR of <60 mL/min/1.73 m^2^. Patients with acute-on-chronic kidney disease, those unwilling to comply with long-term follow-up procedures, and those with active malignancy, autoimmune/inflammatory disorders, or recent (within the past 3 months) major infections were excluded. The primary outcome was all-cause mortality over the course of 1 year. While the cause of death was noted whenever possible, inconsistent data availability across patients limited the cause-specific analysis.

For the analysis, patients were categorized within CKD stages 3, 4, and 5 according to their eGFR values at baseline. Clinical and demographic features collected at the time of admission included age, sex, systolic and diastolic blood pressure, pulse rate, blood glucose levels [fasting blood sugar (FBS), random blood sugar, and glycated hemoglobin (HbA1c)], blood urea, serum creatinine, lipid profile (total cholesterol, high-density lipoprotein, low-density lipoprotein, and very low-density lipoprotein), hemoglobin, total white blood cell count, packed cell volume (PCV), serum albumin, calcium, phosphorous, and alkaline phosphatase (ALP). Baseline eGFR was calculated using the following formula: eGFR = 175 × (serum creatinine)^−1.154^ × (age)^−0.203^ × (0.742 if female) × (1.212 if Black) [13]. Left ventricular ejection fraction was measured with echocardiograms.

For biomarker analysis, 4 mL of peripheral blood was collected from the vein in the antecubital fossa of the forearm using EDTA-coated vacutainer tubes. Blood samples were subjected to centrifugation at 2500 rpm for 10 min, after which plasma samples were extracted and stored in a deep freezer at −80 °C. The plasma samples were subsequently evaluated using the Bio-Plex 200 System (Bio-Rad, Hercules, CA, USA) and the Human Magnetic Luminex Assay Kit (L130811; R&D Systems, Minneapolis, MN, USA) to measure the levels of lipocalin-2, RAGE, TIMP-1, osteopontin, and TFF-3. All laboratory measurements were performed in a blinded manner and the investigators performing the assays were unaware of patient details.

The normality of the data was assessed using the Kolmogorov–Smirnov test for continuous variables. Continuous variables were summarized as mean ± standard deviation or median [interquartile range (IQR)] and categorical data were expressed as numerical frequencies with percentages. Differences in categorical variables between groups were evaluated using the chi-square test. Parametric or nonparametric tests were applied according to the distribution of the data. Differences in continuous variables between groups were analyzed using the Mann–Whitney U test or independent-samples t-test. Pearson or Spearman rank correlation was used to assess associations between variables. Receiver operating characteristic (ROC) curves were plotted to measure the ability of the protein biomarkers to detect mortality. All statistical analyses were performed with SPSS 16.0 (SPSS Inc., Chicago, IL, USA). All p-values were two-sided and p < 0.05 was considered significant. Patients were followed for a duration of 12 months based on electronic hospital records and monthly telephone calls to assess mortality status. No formal sample size calculation was performed due to the exploratory nature of this pilot study.

## 3. Results

### 3.1. Baseline characteristics of study participants

The mean age of the study population (n = 83) was 60.47 ± 12.4 years. All baseline characteristics are presented in Table 1. The majority of the participants were categorized as having CKD of stage 4 (n = 30) or stage 5 (n = 31). Furthermore, 68% of the patients had diabetes and 72% had hypertension.

### 3.2. Median biomarker levels in CKD of stages 3 to 5

The median concentrations of lipocalin-2, RAGE, TIMP-1, osteopontin, and TFF-3 were 56.9 (44.72–64.18) ng/mL, 7.1 (4.92–9.56) ng/mL, 46.44 (33.52–47.56) ng/mL, 98.9 (60.2–167.61) ng/mL, and 8.03 (4.87–15.65) ng/mL, respectively. As seen in Table 2, patients with CKD of stage 4 or 5 had higher concentrations of lipocalin-2 (p = 0.001), RAGE (p = 0.001), osteopontin (p = 0.007), and TFF-3 (p = 0.001) compared to stage 3.

### 3.3. Correlations between various biomarkers and eGFR

**Figure 1:**
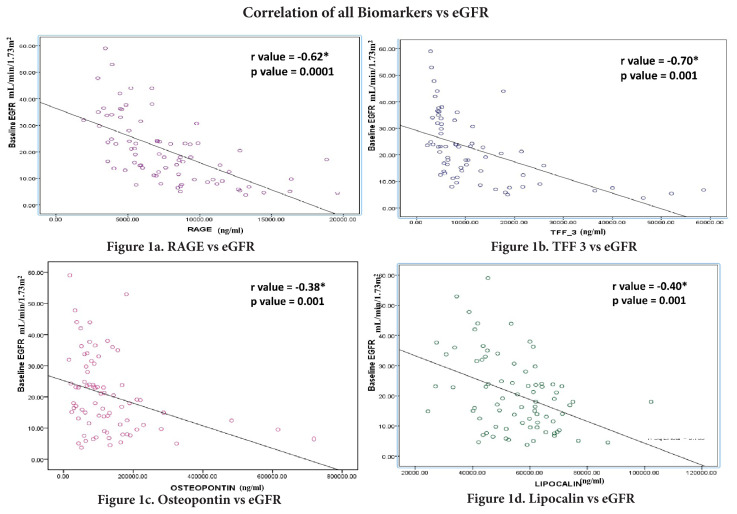
Correlations between various biomarkers and estimated glomerular filtration rate (eGFR). **a)** Negative correlation between receptor for advanced glycation end products (RAGE) and eGFR among study participants. **b)** Negative correlation between trefoil factor 3 (TFF-3) and eGFR among study participants. **c)** Negative correlation between osteopontin and eGFR among study participants. **d)** Negative correlation between lipocalin-2 and eGFR among study participants.

Correlation analysis was performed using data from 77 patients whose samples were free of hemolysis, icterus, or lipemia. Their eGFR values correlated negatively with RAGE (r = −0.62, p = 0.0001; Figure 1a), TFF-3 (r = −0.70, p = 0.001; Figure 1b), osteopontin (r = −0.38, p = 0.001; Figure 1c), and lipocalin-2 (r = −0.40, p = 0.0001; Figure 1d). The concentration of each biomarker was also correlated with various demographic and laboratory characteristics of the study participants (Table 3). There was a negative correlation between age and RAGE (r = −0.25, p = 0.02), whereas SBP had a positive correlation with RAGE (r = 0.26, p = 0.02). FBS had a positive correlation with lipocalin-2 (r = 0.54, p = 0.01). PCV had negative correlations with lipocalin-2 (r = −0.57, p = 0.008) and RAGE (r = −0.56, p = 0.01). There was a negative correlation between hemoglobin and TFF-3 (r = −0.41, p = 0.002). Calcium had negative correlations with both TIMP-1 and TFF-3, whereas albumin had negative correlations with lipocalin-2 and TFF-3. There was a positive correlation between ALP and osteopontin (Table 3).

**Figure 2:**
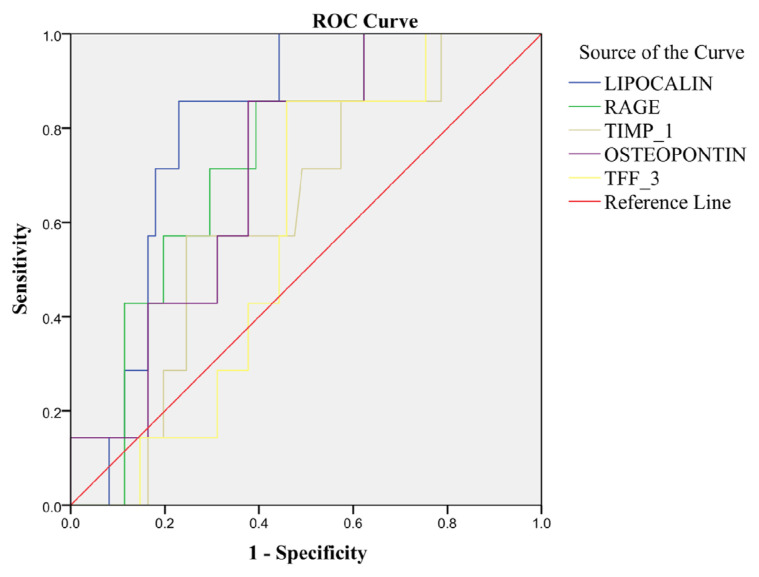
Receiver operating characteristic (ROC) curve of each biomarker plotted to determine the ability to predict mortality among patients with chronic kidney disease. RAGE: Receptor for advanced glycation end products; TIMP-1: tissue inhibitor of metalloproteinase 1; TFF-3: trefoil factor 3.

ROC curves were plotted to determine whether the biomarkers could predict mortality in CKD patients (Figure 2). The cutoff values of these biomarkers for predicting mortality were obtained together with sensitivity and specificity values, as seen in Table 4. The cutoff value predicting mortality was 62.48 ng/mL for lipocalin-2 and 8.5 ng/mL for RAGE.

### 3.4. Association of biomarkers with comorbidities

The associations between the novel renal biomarkers and clinical comorbidities were analyzed using the Mann–Whitney U test (Supplementary Table). Among the biomarkers assessed, RAGE and TFF-3 showed statistically significant associations with family history of CKD (p = 0.033 and p = 0.034, respectively). No other significant associations were found between biomarker levels and comorbidities including diabetes mellitus, hypertension, coronary artery disease, stroke, and chronic heart failure (p > 0.05 for all). These findings indicate that while most biomarker levels are independent of comorbidity status, RAGE and TFF-3 may be influenced by familial or genetic susceptibility to CKD.

## 4. Discussion

This study provides novel insights into the prognostic utility of lipocalin-2 and RAGE in predicting mortality in CKD patients. While these biomarkers have been associated with CKD progression in past studies, our findings suggest that they may also serve as independent predictors of mortality, especially in advanced stages of CKD. This adds a valuable dimension to the clinical relevance of these biomarkers and suggests a potential role for them in risk stratification. We found that lipocalin-2 and RAGE had good predictive value for mortality in CKD. In a study by Isoyama et al., however, it was observed that increased mortality risk was not associated with elevated soluble RAGE [14]. In a study performed with 3386 patients with CKD in Sweden, urine lipocalin-2 concentrations were not independently associated with mortality [15]. However, in that study, a higher concentration of urine lipocalin-2 was independently associated with ischemic atherosclerotic events. Similarly, in a study conducted with 235 patients with ST-segment elevation myocardial infarction in Türkiye, lipocalin-2 values were observed to be higher among patients who died compared to those who survived [16]. A similar finding was obtained in the present study; coronary artery disease was more prevalent among patients who died compared to those who survived. Several studies have explored the link between coronary artery disease and CKD [17]. The risk of sudden cardiac death (SCD) increases as renal function declines. For example, in a study that included patients with implanted cardiac defibrillators and left-ventricular dysfunction, it was observed that for every reduction in eGFR of 10 mL/min/1.73 m^2^, the risk of SCD was 17% higher. The most probable reason for SCD in renal dysfunction is the profound increase in the risk of malignant ventricular arrhythmia [18]. It is not yet known if lipocalin-2 and soluble RAGE play any roles in increasing the risk of arrhythmia in patients with advanced CKD. Besides arrhythmia, other possible causes of death in CKD include autonomic dysregulation [19], electrolyte shifts [20], vascular calcification [21], fibrosis [22], and remodeling of the heart [23]. The roles of lipocalin-2 and RAGE in each of these mechanistic processes need to be explored.

In this study, we found negative correlations between eGFR and renal biomarkers such as lipocalin-2, RAGE, osteopontin, and TFF-3. In a metaanalysis, it was concluded that eGFR had a negative correlation with serum lipocalin-2 [24]. However, urinary lipocalin-2 did not correlate with eGFR. This metaanalysis included data from 17 studies. Although a few of the included studies did not show a correlation between lipocalin-2 and eGFR, the majority of the studies demonstrated a good correlation between the two. Similarly, in a study performed with 49 nondiabetic CKD patients, it was observed that osteopontin levels progressed in a linear fashion as eGFR decreased [25]. In addition, a French study conducted with 150 CKD patients above 40 years of age showed that plasma osteopontin levels were higher in CKD stages 4 and 5 compared to CKD stages 2 and 3 [26]. RAGE has also been demonstrated to increase with worsening CKD [27].

Lipocalin-2 is a protein encoded by the *Lcn2* gene [28]. Preclinical studies have provided robust evidence that lipocalin-2 is a mediator of CKD progression [29]. *Lcn2* inactivation offered protection from interstitial fibrosis as well as glomerular sclerosis after nephron reduction, and *Lcn2* also appears to be a critical transcriptional target of eGFR that could mediate glomerular sclerosis [30]. RAGE-knockout mice were found to have a lesser degree of nephrosclerosis and senile amyloid lesions in comparison to wild-type mice, and there was also a lower degree of oxidative stress and inflammation in RAGE-knockout mice [31]. A deficiency of osteopontin reduced fibrosis of the renal interstitium in mouse models of ischemia/reperfusion injury [32]. Similarly, osteopontin was able to modulate fibrosis in the kidneys in mice injected with angiotensin II [33]. Aldosterone is a factor known to cause renal fibrosis and glomerular injury in association with osteopontin upregulation long before the development of fibrosis. TFF-3 is a peptide secreted in response to injury in renal tubular epithelial cells. The epithelial-to-mesenchymal transition (EMT) is a pathway involved in fibrosis of the renal interstitium. TFF-3 has been hypothesized to participate in the EMT pathway [34].

We observed negative correlations between serum calcium levels and biomarkers including lipocalin-2, RAGE, TIMP-1, and TFF-3. This could be a reflection of worsening CKD as previous studies have shown that hypocalcemia is a well-known electrolyte abnormality in advanced CKD [35]. Interestingly, osteopontin, which has a vital role in bone mineral metabolism in CKD, did not show a correlation with serum calcium, but a positive correlation with alkaline phosphatase was observed. Similar findings were obtained in a study conducted in the U.S. state of Kansas with a total of 92 patients with CKD of stage 5. In CKD patients with low mineral density, ALP was shown to be a suitable marker for both bone turnover and higher mortality. This correlation between osteopontin and ALP was also seen in stage 5 CKD patients in the study conducted by Druck et al. [36]. Osteopontin has a complex relationship with the Ca–PO_4_–vitamin D–parathyroid hormone (PTH) axis. For example, osteopontin-knockout mice were found to have greater bone formation arising due to PTH-induced excess osteoblastic activity [37,38]. Vitamin D has also been shown to result in osteopontin overexpression in animal models [39]. In the present study, the levels of these novel renal biomarkers were largely independent of comorbid conditions such as diabetes mellitus, systemic hypertension, and coronary artery disease. This suggests that the observed elevated biomarker concentrations were likely a reflection of renal dysfunction severity rather than systemic metabolic disturbances. However, the significant association of both RAGE and TFF-3 with family history of CKD was observed, indicating a potential familial or genetic predisposition influencing the upregulation of these markers. Previous studies have demonstrated that the *RAGE* gene and its ligands are linked to genetic susceptibility to CKD progression, particularly through oxidative stress and proinflammatory pathways [9,12]. Teissier et al. reported that RAGE-knockout mice exhibited reduced renal fibrosis and inflammation with aging, suggesting that RAGE activation might be genetically mediated [31]. Similarly, TFF-3 has been implicated as a marker of chronic tubular injury, with elevated levels seen in CKD patients independently of diabetes or cardiovascular disease. The observed association of TFF-3 with family history of CKD in our cohort may reflect heritable alterations in renal tubular repair or fibrotic pathways, consistent with the findings reported by Du et al., who noted a strong link between circulating TFF-3 levels and CKD severity independent of comorbidities [12]. Although lipocalin-2, TIMP-1, and osteopontin did not show significant associations with comorbidities in this analysis, their elevation across CKD stages reinforces their roles as biomarkers of renal injury and disease progression rather than indicators of systemic conditions. These results align with previous studies showing that lipocalin-2 and osteopontin levels rise progressively with declining eGFR independently of diabetes or hypertension [−-26, 27].

This study has limitations including a small sample size and a lack of detailed characterization of CKD patients. For these reasons, prospective studies with significantly larger numbers of patients are needed to corroborate our findings and establish the causality of increased levels of RAGE, lipocalin-2, osteopontin, and TFF-3 in the development of kidney disease. Furthermore, multivariate analysis to adjust for confounders such as age, sex, and comorbidities was not feasible in this study due to the small number of mortality events. This should be considered a limitation, and future studies with larger cohorts should employ Cox regression or logistic models. Another limitation is the lack of longitudinal biomarker measurements. Only baseline levels were assessed, limiting our ability to comment on dynamic changes over time and their associations with outcomes. Demographic and clinical variables were not entirely homogeneous across CKD stages, which may have influenced biomarker levels and mortality risk. This heterogeneity limits the study’s generalizability. Another major limitation of this study is the absence of a prespecified sample size calculation, which may have reduced the statistical power, especially for subgroup comparisons.

It must also be noted that the presence of comorbidities such as diabetes could influence biomarker levels. Although subgroup analysis by diabetic status was considered, the small sample size, particularly for stage 3 CKD, limited the meaningfulness of such stratification. Future studies with larger sample sizes are warranted to control for such confounding variables.

In conclusion, we found that lipocalin-2 and RAGE can predict mortality in patients with CKD. RAGE, lipocalin-2, osteopontin, and TFF-3 were associated with the severity of CKD. Larger studies are needed to establish the relationships between these novel renal biomarkers and the progression of CKD. These markers may be helpful in monitoring the progression of CKD and future studies could explore their utility as pharmacological targets in CKD.

## Supplementary Information

**Supplementary Table:** Association between biomarker levels and comorbidities among CKD patients.

Biomarkers	Comorbidities	U test	Z value	P value
**Lipocalin 2**	Sex	565.00	−1.174	0.240
	T2DM	571.00	−0.715	0.475
	SHTN	541.00	−0.337	0.736
	OBS kidney disease	39.00	−1.153	0.249
	Family history of CKD	124.00	−0.505	0.614
	Stroke	98.00	−0.342	0.732
	CAD	292.00	−1.686	0.92
	CHF	17.00	−0.945	0.345
**RAGE**	Sex	675.00	.00	1.000
	T2DM	635.00	−0.011	0.991
	SHTN	449.00	−1.406	0.160
	OBS kidney disease	63.00	−0.384	0.701
	Family history of CKD	53.00	−2.135	0.033 ^*^
	Stroke	97.00	−0.369	0.712
	CAD	393.00	−0.313	0.754
	CHF	31.00	−0.315	0.753
**TIMP-1**	Sex	525.00	−1.601	0.109
	T2DM	555.00	−0.891	0.373
	SHTN	523.00	−0.546	0.585
	OBS kidney disease	67.00	−0.256	0.798
	Family history of CKD	77.00	−1.584	0.113
	Stroke	68.500	−1.119	0.263
	CAD	297.500	−1.611	0.107
	CHF	10.00	−1.260	0.208
**OPN**	Sex	570.00	−1.121	0.262
	T2DM	595.50	−0.451	0.652
	SHTN	545.50	−0.285	0.776
	OBS kidney disease	65.00	−0.320	0.749
	Family history of CKD	88.00	−1.33	0.183
	Stroke	83.00	−0.73	0.461
	CAD	377.50	−0.524	0.601
	CHF	32.00	−0.270	0.787
**TFF-3**	Sex	484.00	−0.532	0.595
	T2DM	463.00	−0.270	0.787
	SHTN	309.00	−1.867	0.062
	OBS kidney disease	57.00	−0.295	0.787
	Family history of CKD	47.00	−2.091	0.034 ^*^
	Stroke	50.00	−0.553	0.580
	CAD	327.00	−0.049	0.961
	CHF	28.00	−0.259	0.866

## Figures and Tables

**Table 1:** Baseline characteristics of study participants.

Characteristic	Frequency (%) or Mean ± SD (N = 83)
Age (years)	60.47 ± 12.49
Males	54 (65.1)
Etiology	
Contrast-induced Nephropathy	5 (6)
Diabetic nephropathy	10 (12.2)
Comorbidities	
Type 2 diabetes mellitus	57 (68.7)
Systemic hypertension	60 (72.3)
Obstructive kidney disease	2 (2.4)
Family history of CKD	4 (4.8)
Hyperlipidemia	5 (6)
Mineral bone disorder	15 (18.1)
Stroke	3 (3.6)
Coronary artery disease	13 (15.7)
Congenital heart failure	1 (1.2)
Hyperthyroidism	3 (3.6)
Anemia	16 (19.3)
Medication history	
ACE-inhibitors	2 (2.4)
Native medicines	3 (3.6)
Vegetarian diet	48 (57.8)
Symptoms	
Dyspnea	15 (18.1)
Pedal edema	23 (26.7)
Fatigue	27 (32.5)
Itching	13 (15.7)
Bone and joint pain	24 (28.9)
Drowsiness	16 (19.3)
Vomiting	18 (21.7)
Renal cortical cysts	6 (7.2)
Renal echoes	7 (8.4)
Vitals	
Systolic blood pressure (mmHg)	142.44 ± 27.13
Diastolic blood pressure (mmHg)	84.5 ± 13.23
Pulse rate (beats/min)	89.23 ± 17.33
Laboratory investigations	
FBS (mg/dL)	169.35 ± 110.57
HbA1c (%)	7.59 ± 3.05
Urea (mg/dL)	85.6 ± 51.07
Creatinine (mg/dL)	3.82 ± 2.53
Total cholesterol (mg/dL)	138.80 ± 54.23
HDL (mg/dL)	35.2 ± 12.16
LDL (mg/dL)	89.8 ± 35.71
VLDL (mg/dL)	24.8 ± 13.82
Hemoglobin (g/dL)	9.7 ± 2.1
WBC (cells/mm^3^)	7798.00 ± 4539.00
PCV (%)	31.6 ± 5.73
Albumin (g/dL)	3.47 ± 0.51
Calcium (mg/dL)	9.2 ± 3.44
Phosphorous (mg/dL)	4.74 ± 5.42
ALP (IU/L)	1.06 ± 48.46

SD: Standard deviation; CKD: chronic kidney disease; FBS: fasting blood sugar; HbA1c: glycated hemoglobin; HDL: high-density lipoprotein; LDL: low-density lipoprotein; VLDL: very low-density lipoprotein; WBC: white blood cell count; PCV: packed cell volume; ALP: alkaline phosphatase.

**Table 2:** Levels of biomarkers across CKD stages among patients.

Biomarker	Study population (n = 77)#	CKD 3 (n = 18)	CKD 4 (n = 28)	CKD 5 (n = 31)	p-value
Lipocalin-2 (ng/mL)	55.39 ± 14	43.83 ± 9.3	58.78 ± 14.9	57.91 ± 12.27	0.001^*^
RAGE (ng/mL)	7.77 ± 3.76	4.6 ± 1.8	7.4 ± 3.2	9.7 ± 3.8	0.001^*^
TIMP-1 (ng/mL)	41.15 ± 9.85	41.74 ± 9.69	41.08 ± 10.90	40.02 ± 9.4	0.87
Osteopontin (ng/mL)	134.05 ± 122.80	80.31 ± 46.37	101.91 ± 66.52	178 ± 162.38	0.007^*^
TFF-3 (ng/mL)	12.18 ± 11.72	5.76 ± 3.6	9.25 ± 5.6	19.52 ± 1.6	0.001^*^

# The sample size of the original cohort was 83. Out of 83 samples collected, 3 were hemolyzed, 2 were icteric, and 1 was lipemic.

Those 6 samples were excluded from the analysis. CKD: Chronic kidney disease; RAGE: receptor for advanced glycation end products; TIMP-1: tissue inhibitor of metalloproteinase 1; TFF-3: trefoil factor 3. Levels of biomarkers are expressed as mean ± standard deviation.

**Table 3:** Correlations between biomarkers and patient characteristics.

	Lipocalin-2 (ng/mL)	RAGE (ng/mL)	TIMP-1 (ng/mL)	OPN (ng/mL)	TFF-3 (ng/mL)

Age (years)					
r value	−0.062	−0.250*	0.064	−0.041	0.022
p value	0.592	0.028	0.581	0.725	0.861

SBP (mmHg)					
r value	0.071	0.263*	0.003	0.095	0.115
p value	0.557	0.028	0.979	0.432	0.380

FBS (mg/dL)					
r value	0.545*	0.140	0.163	−0.106	0.425
p value	0.019	0.578	0.518	0.674	0.114

Urea (mg/dL)					
r value	0.087	0.538**	−0.232	0.255*	0.526**
p value	0.477	0.001	0.055	0.034	0.001

Creatinine (mg/dL)					
r value	0.353**	0.707**	0–.194	0.341**	0.692**
p value	0.002	0.001	0.091	0.002	0.001

Hemoglobin (g/dL)					
r value	−0.220	−0.357**	−0.045	−0.240	−0.407**
p value	0.083	.004	0.726	0.058	0.002

PCV (%)					
r value	−0.573**	−0.564**	−0.193	−0.205	−0.235
p value	0.008	0.010	0.415	0.386	0.347

Calcium (mg/dL)					
r value	−0.614**	−0.612**	−0.454*	−0.279	−0.595**
p value	0.001	0.001	0.017	0.159	0.003

ALP (IU/mL)					
r value	0.168	0.194	0.162	0.459*	0.311
p value	0.444	0.376	0.460	0.027	0.182

RAGE: Receptor for advanced glycation end products; TIMP-1: tissue inhibitor of metalloproteinase 1; OPN: osteopontin; TFF-3: trefoil factor 3; SBP: systolic blood pressure; FBS: fasting blood sugar; PCV: packed cell volume; ALP: alkaline phosphatase;

* significant difference at p < 0.05;

** highly significant difference at p < 0.01.

Correlations between renal biomarkers and patient characteristics are expressed using the Spearman correlation coefficient (r).

**Table 4:** Cutoff values for prediction of mortality by the biomarkers together with sensitivity and specificity.

Biomarkers	AUC	Asymptotic 95% Confidence Interval	P value	Cut off (ng/mL)	Sensitivity %	Specificity %
Lipocalin (ng/mL)	0.803	(0.687–0.919)	0.009^**^	62.48	85.7	77
RAGE (ng/mL)	0.735	(0.580–0.891)	0.043^*^	8.50	71.4	70.5
TIMP-1 (ng/mL)	0.615	(0.430–0.799)	0.323	-	-	-
Osteopontin (ng/mL)	0.712	(0.550–0.873)	0.068	-	-	-
TFF-3 (ng/mL)	0.578	(0.417–0.740)	0.499	-	-	-

AUC: Area under the curve; CKD: chronic kidney disease; RAGE: receptor for advanced glycation end products; TIMP-1: tissue inhibitor of metalloproteinase 1; OPN: osteopontin; TFF-3: trefoil factor 3.
